# Revisiting the Single Cell Protein Application of *Cupriavidus necator* H16 and Recovering Bioplastic Granules Simultaneously

**DOI:** 10.1371/journal.pone.0078528

**Published:** 2013-10-24

**Authors:** Balakrishnan Kunasundari, Vikneswaran Murugaiyah, Gurjeet Kaur, Frans H. J. Maurer, Kumar Sudesh

**Affiliations:** 1 School of Biological Sciences, Universiti Sains Malaysia, Penang, Malaysia; 2 School of Pharmaceutical Sciences, Universiti Sains Malaysia, Penang, Malaysia; 3 Institute for Research in Molecular Medicine, Universiti Sains Malaysia, Penang, Malaysia; 4 Department of Chemistry, Polymer & Materials Chemistry, Lund University, Lund, Sweden; Laurentian University, Canada

## Abstract

*Cupriavidus necator* H16 (formerly known as *Hydrogenomonas eutropha*) was famous as a potential single cell protein (SCP) in the 1970s. The drawback however was the undesirably efficient accumulation of non-nutritive polyhydroxybutyrate (PHB) storage compound in the cytoplasm of this bacterium. Eventually, competition from soy-based protein resulted in SCP not receiving much attention. Nevertheless, *C. necator* H16 remained in the limelight as a producer of PHB, which is a material that resembles commodity plastics such as polypropylene. PHB is a 100% biobased and biodegradable polyester. Although tremendous achievements have been attained in the past 3 decades in the efficient production of PHB, this bioplastic is still costly. One of the main problems has been the recovery of PHB from the cell cytoplasm. In this study, we showed for the first time that kilogram quantities of PHB can be easily recovered in the laboratory without the use of any solvents and chemicals, just by using the cells as SCP. In addition, the present study also demonstrated the safety and tolerability of animal model used, Sprague Dawley given lyophilized cells of *C. necator* H16. The test animals readily produced fecal pellets that were whitish in color, as would be expected of PHB granules. The pellets were determined to contain about 82-97 wt% PHB and possessed molecular mass of around 930 kg/mol. The PHB granules recovered biologically possessed similar molecular mass compared to chloroform extracted PHB [950 kg/mol]. This method now allows the production and purification of substantial quantities of PHB for various experimental trials. The method reported here is easy, does not require expensive instrumentation, scalable and does not involve extensive use of solvents and strong chemicals.

## Introduction

Despite their excellent stability, durability, weight saving and insulating properties, plastics derived from petrochemicals are fast acquiring a negative image through their ubiquitous persistence in the environment, posing a threat to our natural habitats [1]. Besides this, the continuous rise in the volume of plastics produced every year places increasing demands on the world’s finite natural resources, and exerts pressure on its fragile ecosystems [2-4]. Hence, plastic manufacturers and researchers are joining forces to develop superior bio-based polymers with the view of introducing commercially viable new materials that will find consumer acceptance. 

 Polyhydroxyalkanoates (PHAs) are being extensively investigated as a potential candidate to replace synthetic polymers mainly due to use of renewable resources, biodegradability as well as their tunable mechanical and thermal properties by copolymerization [5,6]. Though PHAs can be technically specified to meet various applications, high production costs is a serious deterrent to their successful commercialization [2,7]. Feedstock for the biosynthesis of PHA and the subsequent recovery processes are the major cost absorbing factors which account for the overall high production cost [8]. Since PHA has been produced using microbial fermentation mostly with *Cupriavidus necator* H16 (previously known as *Hydrogenomonas eutropha*) [2,8,9], recent studies have been geared toward identifying renewable and inexpensive carbon sources in order to bring down the overall costs [10-13]. Nevertheless, the price of PHA also hinges largely on downstream processing; hence, the importance of developing an economical and efficient recovery processes cannot be overestimated [14,15]. The currently available methods are not only expensive, but they may also be hazardous to the environment because of the use of solvents and strong chemicals. A comprehensive review on the various PHA recovery methods that have evolved is available elsewhere [14]. 

 In recent decades, the bacterium *C. necator* H16 has been well known among researchers mainly for its ability to synthesize PHA. Nevertheless, the initial interest in this wild type strain was not for its PHA synthesizing ability, but for its nutritive value [16,17]. In the 1970s, much work was devoted to utilizing *C. necator* H16 as a source of single cell protein (SCP) for animal feed owing to its high protein content and quality [16]. Schlegel and Lafferty estimated that the gross composition of most bacterial cells would average 50% protein, 15% nucleic acids and 20% cell wall substances [18]. Almost 93% of bacterial protein was found to be digestible by animals in a study by Calloway and Kumar [16]. Analysis of the nutritive values of the bacterial cells showed that the concentrations of certain important amino acids were similar to those found in casein.

 Though the nutritive aspects of the cells were found promising to be developed as SCP, Waslien and Calloway [17] encountered an obstacle in that the *C. necator* H16 cells also accumulated poly(3-hydroxybutyrate), PHB, a biopolymer of the PHA family. Most of the PHB granules consumed by animals were excreted without being absorbed by the digestive system. As PHB does not possess any nutritive value, attempts were made to suppress its synthesis and accumulation in bacterial cells [18]. 

 There has been a considerable resurgence of interest in recent years to use PHA as a component of animal feed to increase the metabolizable energy content [19-23]. To improve digestibility, the PHA is pre-treated with sodium hydroxide (NaOH) before being fed to animals [21]. Patent literature describes the use of animal feed containing whole bacterial cells with PHA for modulation of the gut flora by delivering short-chain fatty acids such as 3-hydroxybutyric acids [19,24]. Defoirdt et al. [25] reviewed the possible application of PHA as new biocontrol (bacteriostatic) agents for animal production. 

 It is evident from previous studies that PHAs are not toxic and have been used in animal feed [17,20-23]. The *C. necator* H16 cells containing PHB was found to be tolerated by animals. These studies also reported the poor digestibility of PHAs by monogastric animals. However, it is important to highlight that previously, the excretion of PHB in the fecal pellets was the main concern because the primary objective was to increase the metabolized energy of the animal feed through conversion of these energy-rich substances within the nutritional chain [17,20,21,23]. In contrast to previous studies, the present study took advantage of the excretion of undigested PHA to be developed into a method for recovering PHA [26]. 

 The main objective of this study was to develop a novel biological recovery process of PHA that form the basis for a combined synergetic feed and purification process of PHA granules from the lyophilized cells of *C. necator* H16 without extensive use of solvents and strong chemicals. By taking into account the well-documented nutritive aspects of *C. necator* H16 cells, the welfare and tolerability of the animal model (Sprague Dawley) given lyophilized cells of *C. necator* H16 containing PHB as sole diet source were evaluated for study intervals of 1, 2 and 4 weeks. 

## Materials and Methods

### Bacterial strain

*Cupriavidus necator* H16 (ATCC 17699) was used throughout this study. The bacterium was maintained on nutrient rich (NR) agar containing 14 g/L of bacteriological agar powder. 

### PHB biosynthesis in 2000-L fermenter

PHB biosynthesis was carried out in one-stage fed-batch cultivation of *C. necator* H16 in a 2000 L fermenter (SIRIM Bioplastic Pilot Plant facilities, Selangor, Malaysia). The cells were cultivated according to the method described by Budde et al. [27] with some modifications. The preculture was initiated by streaking *C. necator* H16 on NR agar plate and incubated for 12 h at 30°C. Two flasks containing 50 mL NR broth were inoculated with two loopfuls of bacterial cells each and cultivated at 30°C, 200 rpm for 7 h (OD_600nm_ ~ 4.0). Aliquots of 6 mL [3% (v/v)] of the preculture was transferred into 9 flasks containing 194 mL of NR medium each and cultivated at 30°C, 200 rpm for 7 h until the cells were in their mid-exponential growth phase (OD_600nm_ ~ 4.0). Then, 1.7 L [10% (v/v)] of the respective precultures was inoculated into 15.3 L of MM containing 20 g/L fructose, 1 g/L of CO(NH_2_)_2_, 0.23 g/L of MgSO_4_·7H_2_O as well as 1 ml/L of trace elements [28] in a 20 L fermenter at 30°C and 200 rpm. The preculture was transferred into 133 L of MM in a 200 L fermenter when the OD_600nm_ reached approximately 12.0. Experiment conditions and medium formulation were maintained as in 20 L fermenter except with carbon source, fructose was substituted with 10 g/L of crude palm kernel oil (CPKO). After 7 h cultivation (OD_600nm_~ 12), 100 L [10% (v/v)] of preculture was inoculated into 900 L of MM. Total concentrations of 6 g/L of CO(NH_2_)_2_ and 30 g/L of CPKO were added during the fermentation. The medium was also supplemented with 0.46 g/L of MgSO_4_·7H_2_O (0.23 g/L during inoculation and also at 12 h of cultivation) and 1.5 ml/L of trace elements (1 mL/L during inoculation and 0.5 mL/L at 12 h of cultivation). The stirrer speed was increased from 200 to 350 rpm to maintain the level of dissolved O_2_ at 40% saturation and the pH was adjusted to 7.0 by using either 3 M HCl or NaOH. After 20 h of cultivation, the cells were harvested by a continuous centrifuge (Alfa Laval) at 5000 × *g* for 10 min at 4° C. The cell pellets were then frozen at -20°C for 48 h before freeze-drying for three days. 

Tolerability and safety of rats fed with the lyophilized cells of *C. necator* H16 as sole diet source 

### Ethics statement

Approval for this study was obtained from Animal Ethics Committee, Universiti Sains Malaysia (AECUSM, Approval No: USM/Animal Ethics Approval/ 2011/ (62) (294). The handling and use of animals was in accordance with the institutional guidelines. 

### Animals and housing

Sprague Dawley (SD) rats of both sexes between 8 to 12 weeks weighing initially 150-200 g were used in this study. The animals were obtained from Animal Research and Service Centre (ARASC), Universiti Sains Malaysia. The animals were housed individually and allowed to acclimatize for a week prior to experimentation. The animals were maintained on a 12 h light/dark cycle at 25°C and relative humidity of 50-60%. The rats were allowed free access to commercial food pellets (Gold Coin; Penang, Malaysia) and drinking water (tap water). 

### Experimental design

The evaluations on the tolerability and safety of rats given lyophilized cells of *C. necator* H16 as sole diet source were carried out for study periods of 7, 14 and 28 days. The rats were randomly assigned to test and control groups, consisting of 6 males and 6 females each. The animals were fasted overnight at the beginning and end of the experiments. The initial weights of the rats were measured and the body weight changes were monitored twice a week. The test group received fixed amount (15 g/day/animal) of only lyophilized cells of *C. necator* H16 containing PHA in the form of flakes while the control group was provided with fixed amount of commercial food pellets (15 g/day/animal) as their feed [29]. The amount of feed given for both test and control groups were adjusted individually according to the daily consumption from second day onwards. Throughout the experiments, water was given *ad libitum*. Food intake and water consumption were monitored and recorded. General observations were conducted daily for possible clinical signs amongst test animals. Detailed clinical observations were carried out once a week to determine any unusual signs such as changes in skin, fur or response to handling. At the end of the study period, all animals were lightly anaesthetized with diethyl ether and terminal blood samples were withdrawn via cardiac puncture for haematological and blood biochemical analysis. Thereafter, these animals were sacrificed for gross and histopathological examination. The fecal pellets excreted by animals given only lyophilized cells of *C. necator* H16 containing PHA were collected every week, weighed and dried overnight in an oven at 60°C to eliminate moisture. An aliquot of the raw fecal pellets was sub-sampled and directly subjected to viable cell count without being exposed to heat.

### Haematological evaluation

Blood samples were collected as described above and transferred into EDTA containing tubes. The following haematological parameters were assessed using an automated haematology analyzer (Cell-Dyn 3500, Abbott Diagnostics, USA): red blood cells count, haemoglobin, haematocrit, mean cell volume, mean cell haemoglobin, mean cell haemoglobin concentration, red cell distribution width, platelet count, mean platelet volume, total white blood cells count, lymphocytes, monocytes, neutrophils, eosinophils and basophils. 

### Blood biochemistry evaluation

Blood biochemical evaluation was conducted on the serum samples using an automated biochemistry analyzer (Architect CI8200, Abbott Diagnostics, USA). The samples were assayed for renal functions tests (calcium, chloride, sodium, potassium, phosphate, creatinine, urea and uric acid), liver function tests (alanine aminotransaminase [ALT], aspartate aminotransferase [AST], alkaline phosphatase [ALP], total protein, albumin and total bilirubin), serum lipids (total cholesterol and triglyceride) and glucose. 

### Histopathological evaluation

The following organs (liver, kidney, adrenal, spleen, stomach, small intestine, lung, heart, testis and ovary) were removed and cleaned with saline solution [0.90% (w/v) NaCl]. Their wet weights were taken and subsequently tissues were fixed in 10% v/v neutral buffered formalin. Liver and kidney samples of rats given lyophilized cells of *C. necator* H16 and controls were subjected to histopathological evaluations. Tissue samples were processed and embedded in parafﬁn. Sections of approximately 3 μm thickness were cut followed by staining with haematoxylin-eosin and viewed under bright field microscopy. 

### Estimation of viable cell count

Microbial population in the fecal pellets was characterized by standard spread plate methodology. Sample weighing 1 g was added into 9 mL of sterile distilled water and was agitated vigorously. The resulting suspension was serially diluted and inoculated on nutrient agar plates. The plates were incubated at 30°C for 24 h. The results were expressed as colony forming units per mL (cfu/mL).

### Polymer extraction using chloroform (solvent extraction)

PHA polymers accumulated in the cells were extracted by refluxing 1 g of lyophilized cells in 100 mL chloroform for 4 h at 60°C. The resulting chloroform extract was cooled to room temperature and residual biomass was removed by filtration. The filtrate was concentrated to 10 mL and then precipitated by adding the concentrated extract drop-wise into 100 mL of vigorously stirred chilled methanol. The resulting white polymer material was recovered by centrifugation at 10000 × *g* for 10 min and air dried overnight.

### Analytical procedures

#### Protein quantification

The protein content of lyophilized cells and fecal pellets were determined as described by De Mey et al. [30]. A total of 100 mg of sample was suspended in 5 mL of 40 mM Na_3_PO_4_ buffer and the sample solution was sonicated (Virsonic Cell Disrupter, Gardiner, USA) for 2 min with 10 s interval at 4°C. Subsequently, 5 mL of 2 N NaOH was added to the test solution and boiled for 10 min, followed by immediate cooling on ice. Protein release was determined using the Bradford method with bovine serum albumin as the standard [31]. Five millilitres Bradford reagent was added to 100 µL of sample and incubated for 15 min at room temperature. Absorbance was measured at 595 nm using a Jenway 6505 UV/Vis. spectrophotometer (Jenway, UK).

#### Gas chromatography (GC)

The PHB content of lyophilized cells and fecal pellets of the treated group were determined according to standard methods [32] using Shimadzu GC-2010 (Shimadzu, Japan) equipped with AOC-20i Auto-Injector. Approximately 15 mg of samples were subjected to methanolysis in the presence of 2 mL of 85% (v/v) methanol acidified with 15% (v/v) sulfuric acid and 2 mL of chloroform for 140 min at 100°C. The resulting methyl esters were then quantified with caprylic methyl ester as an internal standard. The column temperature was initiated at 70°C and then increased to 280°C in a continuous step of 14 °C/min. 

#### Size exclusion chromatography (SEC)

Molecular mass data were obtained by size exclusion chromatography (SEC) analysis at 40°C using the Agilent 1200 GPC (Agilent Technologies, USA) system fitted with a refractive index detector (RID) and Shodex K-806M and K-802 columns. Chloroform was used as the eluent at a flow rate of 0.8 mL/min. Sample concentration of 1.0 mg/mL was applied after being filtered using 0.45 µm PTFE filter (Sartorius, Germany). Polystyrene standards with a low polydispersity were used to construct a calibration curve. 

#### Differential scanning calorimetry (DSC)

Differential scanning calorimetry (DSC) data were recorded in the temperature range of −120 to 200°C on a Perkin-Elmer Pyris 1 (PerkinElmer Inc., USA) instrument equipped with a cooling accessory under a nitrogen flow rate of 20 mL/min. Samples were encapsulated in aluminum pans and heated from 25 to 200°C at a heating rate of 20 °C/min (first heating scan). The melt samples were then maintained at 200°C for 1 min and followed by rapid quenching to −120°C. They were heated again from −120 to 200°C at a heating rate of 20 °C/min (second heating scan). The glass transition temperature (*T*_g_) was taken as the midpoint of the heat capacity change. The melting temperature (*T*_m_) and the heat of fusion (Δ*H*_m_) were determined from the DSC endotherm. 

#### Chemical based-recovery of PHA

The lyophilized cells which were obtained from 2000 L fermenter experiment were also treated with various chemicals at different concentrations to digest non polymeric cellular materials (NPCM) for the recovery of P(3HB). The cells were ground to powder and a total of 1 g of cells was resuspended in 100 mL of respective solutions (Table 1). Digestion was carried out for 1 h at 30°C with agitation speed of 250 rpm. The solution was then centrifuged at 8000 × *g* for 15 min. The supernatant was discarded and the pellet was then re-suspended in 100 mL of distilled water followed by the centrifugation. This step was repeated twice. At the end, the pellet was transferred to a clean test tube which had been dried and pre-weighed. The resulting granules were then air-dried for 48 h. The final weight of the purified P(3HB) granules was recorded. All experiments were conducted in triplicate to examine reproducibility and the average values are reported. Quantification of purified PHA was carried out, by GC analysis, in a similar manner as for freeze-dried cells. Polymer purity and recovery yield were determined as described by Choi and Lee [8]. 

**Table 1:** PHA recovery by digestion with various chemicals.

Sample	Chemicals	Concentrations
P(3HB)_A	HCl	0.05 M, 0.1 M, 0.2 M, 0.5 M
P(3HB)_B	NaOH	0.05 M, 0.1 M, 0.2 M, 0.5 M
P(3HB)_C	SDS	0.1 g/L, 2.5 g/L, 5 g/L, 10 g/L
P(3HB)_D	NaOCl	5%, 10%, 15%, 20%
P(3HB)_E	Dispersions of CHCl_3_ and NaOCl	50 mL of CHCl_3_:50 mL of NaOCl (5%, 10%, 15%, 20%)

HCl, hydrochloric acid; NaOH, sodium hydroxide; SDS, sodium dodecyl sulphate; NaOCl, sodium hypochlorite; CHCl_3,_ chloroform

### Statistical analysis

Experimental data were presented as mean ± standard deviation (SD) and analyzed using SPSS^®^ 17.0 software (SPSS Inc., Chicago, IL). The data on organ weights, haematology, and biochemistry parameters were analyzed for normality by Shapiro–Wilk test, homogeneity of variance by Levene test and statistical significance by t-test. The data was considered as significantly different at *p* value <0.05. When data were not normally distributed from at least 1 group, a Mann–Whitney U-test was performed. Tukey’s HSD (Honestly Significant Difference) test was used in conjunction with one-way analysis of variance (ANOVA) for single step multiple comparisons.

## Results

### Biosynthesis of PHB

In order to minimize the variability of biomass used for the tolerability and safety evaluations, a large amount of cells containing PHA was cultivated using a 2000 L fermenter. A total of 31 kg of dry cells was obtained. The cells contained a moderate amount of PHB content, 39 ± 1 wt%. The crude protein content of the lyophilized cells was determined to be 43 ± 3 wt%. 

Tolerability and safety of rats fed with the lyophilized cells of *C. necator* H16 as sole diet source for 7, 14, and 28 days

Initial evaluation on the tolerability and safety of rats given lyophilized cells of *C. necator* H16 as sole diet source was conducted for 28 days. The subsequent study intervals of 7 and 14 days were chosen as poor weight gain was observed with animals fed with *C. necator* H16 for 28 days indicating that the animals were not receiving enough nutrients for optimal growth. The results are presented in the following sub-sections.

### Gross observation, body weight, and food and water consumption

**Figure 1:**
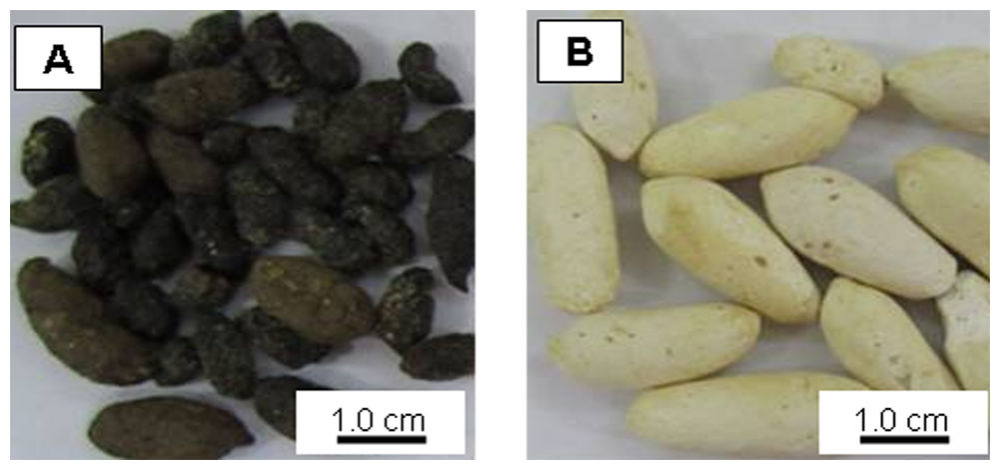
The appearance of the faeces of the (A) Control animals and (B) Test animals fed with lyophilized cells of *C. necator* H16 containing 39 wt% PHB.

**Figure 2:**
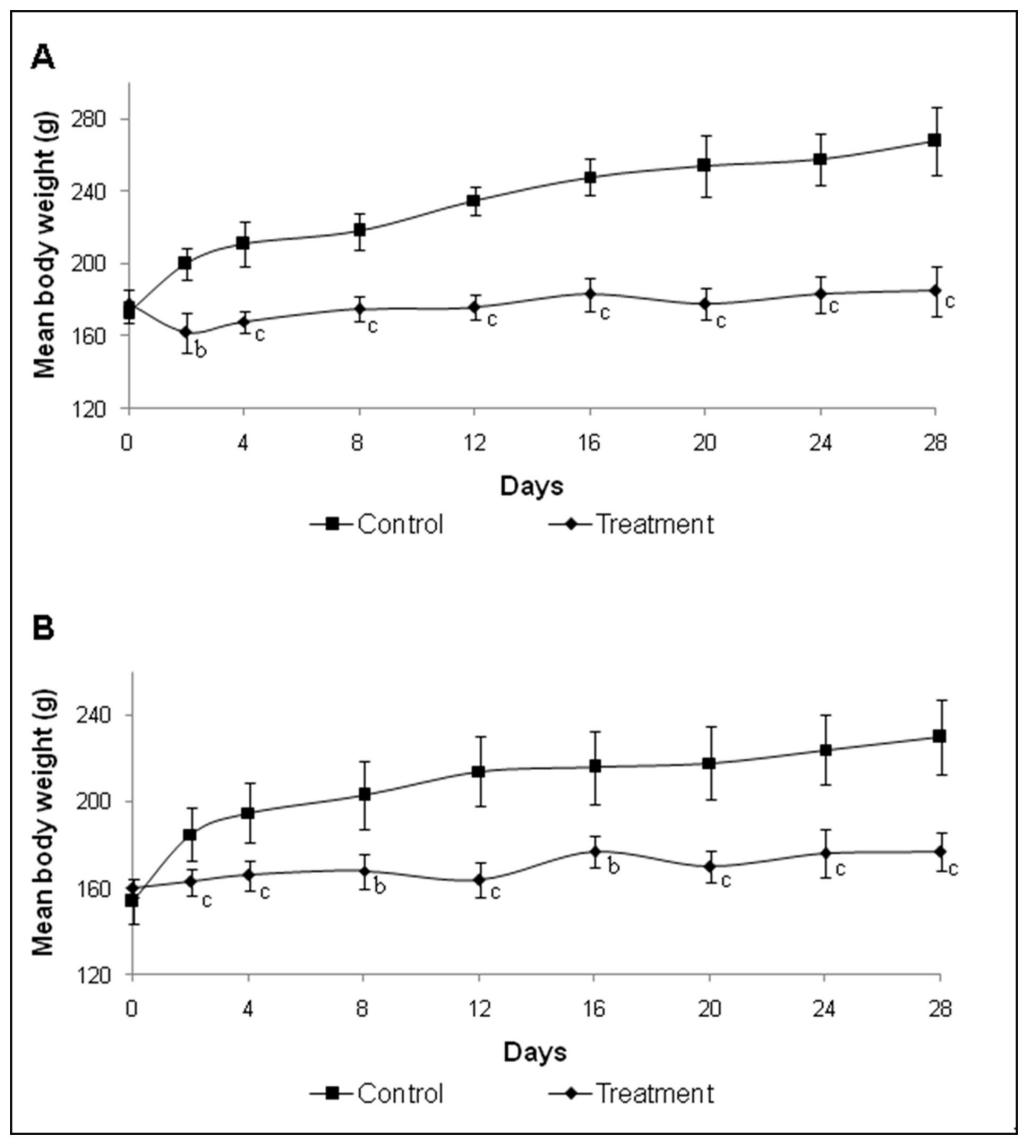
Growth curves of the bacterial cells diet fed- and control groups during 28 days of study period (A) Male and (B) Female. The values are expressed as mean ± SEM (*n*=6 per group). *P-values were calculated by using the t-test. ^a^P<0.05; ^b^P<0.01; ^c^P<0.001 as compared to control group.

No mortality was observed and the animals tolerated well the bacterial cells diet throughout the study. No abnormalities in terms of behaviour were observed, however diarrhoea was noticed during terminal blood collection with rats given bacterial cells diet for 7 days. Interestingly, all the animals on the bacterial cells diet excreted white-colored fecal pellets (WFP) that had low odour, while animals in the control group produced the normal dark-colored fecal pellets (DFP) (Figure 1). Animals receiving bacterial cells diet had significantly poor weight gain than those fed with commercial food regardless of feeding duration. Figure 2 illustrates the growth curves of the male and female rats of both test and control groups during 28 days of study. Similar growth patterns were obtained from 7 and 14 days of feeding experiments (data not shown). 

The average food consumption of the bacterial cells diet fed groups was 11-25 g/day, similar to those of the control animals (12-25 g/day). The average daily water intake of the bacterial cells diet fed animals was approximately two-fold higher than the control groups. Similar trends were noticed in the amounts of daily food and water intake between test and control groups during 7 and 14 days of study. The total amount of bacterial cells diet consumed by the test groups was 0.86 kg (7 days), 2.35 kg (14 days) and 4.22 kg (28 days). The PHA content of WFP was quantified to be in a range of 87-90 wt%. PHA was not detected in DFP from the control animals. The ratio of the WFP to the amount of bacterial cells diet consumed showed that 40-47% of the feed was discharged in the excrement.

### Haematological evaluation

The haematological findings of the bacterial cells diet fed and control groups of both sexes are summarized in Table 2. Significant increases in red blood cells, haemoglobin and haematocrit were noted in animals of both sexes fed with bacterial cells diet for 7 and 28 days, compared to their respective controls. In contrast, these parameters were found to be unaffected in rats given bacterial cells diet for 14 days. In general, the white blood cell counts of animals receiving bacterial cells diet were noticed to be higher, however, the difference was only significant in female rats fed with bacterial cells diet for 7 days (*p*<0.05). The differential count revealed that the subset of neutrophil granulocytes in bacterial cells diet fed animals for 14 days were significantly higher than control animals receiving commercial pellets (male: *p*=0.041; female: *p*=0.001). Similar finding was found in female rats given the bacterial cells diet for 7 days (*p*=0.039). A significant decrease in the subset of monocytes (*p*= 0.036) was recorded in females given bacterial cells diet for 28 days.

**Table 2:** Haematological parameters of bacterial cells diet fed and control rats (7, 14 and 28 days experiments).

Group	7 days		14 days		28 days	
	Control	Test	Control	Test	Control	Test
**Males**						
Red blood cell ( x10^12^/L)	**7.47 ± 0.2**	**9.13 ± 0.6^c^**	7.45 ± 0.5	8.06 ± 0.8	8.20 ± 0.3	**9.78 ± 0.5^c^**
Haemoglobin ( g/dL)	**14.7 ± 0.5**	**16.9 ± 0.7^b^**	14.2 ± 0.6	14.8 ± 2.2	14.3 ± 0.6	**16.8 ± 1.1^b^**
Haematocrit (%)	**63.1 ± 2.5**	**72.7 ± 3.8^c^**	65.1 ± 3.2	68.8 ± 4.8	64.1 ± 3.4	**76.2 ± 3.7^c^**
White blood cells ( x10^3^/µL)	14.0 ± 3.5	15.1 ± 3.7	13.6 ± 3.0	13.2 ± 3.7	15.6 ± 1.0	16.6 ± 3.5
Neutrophils ( x10^3^/µL)	3.31 ± 1.1	4.83 ± 1.6	2.46 ± 1.4	**4.19 ± 1.0^a^**	4.71 ± 1.4	6.09 ± 3.8
Lymphocytes ( x10^3^/µL)	9.74 ± 2.2	9.10 ± 2.9	9.65 ± 1.4	8.17 ± 3.0	9.61 ± 1.3	9.69 ± 2.6
Monocytes ( x10^3^/µL)	0.22 ± 0.3	0.30 ± 0.9	0.86 ± 0.3	0.51 ± 0.3	0.54 ± 0.4	0.34 ± 0.4
Eosinophils ( x10^3^/µL)	0.47 ± 0.1	0.34 ± 0.2	0.11 ± 0.1	0.05 ± 0.1	0.29 ± 0.1	0.19 ± 0.2
Basophils ( x10^3^/µL)	0.24 ± 0.2	0.48 ± 0.5	0.48 ± 0.3	0.27 ± 0.2	0.39 ± 0.1	0.32 ± 0.3
**Females**						
Red blood cell ( x10^12^/L)	**7.34 ± 0.3**	**8.18 ± 0.2^c^**	7.92 ± 0.4	7.99 ± 0.9	**7.68 ± 0.4**	**9.10 ± 0.5^c^**
Haemoglobin ( g/dL)	**14.8 ± 0.6**	**16.3 ± 0.5^b^**	14.6 ± 0.4	14.9 ± 1.6	**14.1 ± 0.6**	**16.5 ± 0.9^c^**
Haematocrit (%)	**63.5 ± 2.2**	**67.5 ± 1.9^b^**	69.1 ± 2.0	67.6 ± 7.3	**63.2 ± 4.5**	**73.6 ± 3.8^b^**
White blood cells ( x10^3^/µL)	**13.4 ± 0.6**	**17.4 ± 4.2^a^**	10.9 ± 2.2	12.8 ± 3.7	12.3 ± 2.7	11.2 ± 2.5
Neutrophils ( x10^3^/µL)	**3.27 ± 0.9**	**5.67 ± 2.3^a^**	**1.46 ± 0.5**	**3.04 ± 0.7^b^**	2.96 ± 1.2	2.10 ± 0.8
Lymphocytes ( x10^3^/µL)	8.97 ± 1.1	10.7 ± 2.2	8.47 ± 1.7	8.55 ± 3.4	8.37 ± 2.8	8.55 ± 2.1
Monocytes ( x10^3^/µL)	0.46 ± 0.2	0.27 ± 0.4	0.59 ± 0.1	0.68 ± 0.6	**0.46 ± 0.3**	**0.13 ± 0.1^a^**
Eosinophils ( x10^3^/µL)	0.40 ± 0.1	0.43 ± 0.2	0.18 ± 0.1	0.11 ± 0.1	0.17 ± 0.1	0.19 ± 0.1
Basophils ( x10^3^/µL)	0.33 ± 0.1	0.28 ± 0.3	0.23 ± 0.1	0.38 ± 0.5	0.30 ± 0.2	0.14 ± 0.1

Values are expressed as means ± SD (n= 6 per group).

*P-values were calculated by using the t-test for normally distributed data and the Mann–Whitney test for non-normally distributed data.

^a^ P<0.05; ^b^P<0.01; ^c^P<0.001 as compared to control group.

### Blood biochemistry evaluation

The results of serum biochemical tests of both bacterial cells diet fed and control animals are summarized in Tables 3 and 4. When compared to their respective control groups, in general, most of the parameters tested were found to be significantly affected in animals receiving bacterial cells diet for 14 days. Statistically significant reduction in serum calcium and increase in urea concentration were recorded with all bacterial cells diet fed animals compared to their respective control groups except for female rats given bacterial cells diet for 28 days. A significant decrease in chloride level was noted in female rats given bacterial cells diet for 14 (*p*=0.001) and 28 (*p*=0.002) days. The sodium concentration of test male rats for 7 (*p*=0.036) and 14 days (*p*=0.040) were observed to be statistically lower than their respective controls. Similar result was obtained with female rats given bacterial cells diet for 28 days (*p*=0.041). Among all tested animals, a statistically lower serum potassium (*p*=0.016) level was only noticed with bacterial cells diet fed females for 7 days. The serum phosphate, creatinine, and uric acid were analyzed to be statistically different in males receiving bacterial cells diet for 14 and 28 days. The phosphate level of females given bacterial cells diet for 14 and 28 days were also found to be significantly lower than animals receiving commercial food. Serum creatinine and uric acid concentrations were statistically higher in female rats fed with bacterial cells diet for 7 and 14 days. 

**Table 3:** Plasma biochemical findings of bacterial cells diet fed and control male rats (7, 14 and 28 days experiments).

Group	7 days		14 days		28 days	
	Control	Test	Control	Test	Control	Test
Calcium (mmol/L)	**2.49 ± 0.1**	**2.20 ± 0.1^c^**	**2.60 ± 0.1**	**2.25 ± 0.1^c^**	**2.58 ± 0.4**	**2.25 ± 0.1^a^**
Chloride (mmol/L)	103.2 ± 1.2	104.0 ± 2.1	**102.17 ± 1.5**	**107.5 ± 2.4^b^**	95.2 ± 13.7	99.5 ± 5.4
Sodium (mmol/L)	**136.5 ± 1.4**	**133.8 ± 2.3^a^**	**142.33 ± 1.0**	**140.83 ± 1.8^a^**	127.7 ± 14.5	135.5 ± 2.7
Potassium (mmol/L)	6.18 ± 0.4	5.73 ± 0.5	4.35 ± 0.2	4.48 ± 0.5	6.03 ± 1.1	6.17 ± 1.3
Phosphate (mmol/L)	2.66 ± 0.2	2.51 ± 0.4	**2.73 ± 0.1**	**2.25 ± 0.3^b^**	**3.00 ± 2.0**	**1.54 ± 0.4^a^**
Creatinine (µmol/L)	35.9 ± 1.3	36.1 ± 1.7	**42.70 ± 1.3**	**45.83 ± 2.0^a^**	**48.5 ± 12.6**	**40.5 ± 1.9^a^**
Urea (mmol/L)	**5.4 ± 0.4**	**13.0 ± 0.7^c^**	**8.00 ± 0.4**	**11.20 ± 2.8^a^**	**7.53 ± 1.0**	**10.3 ± 4.3^a^**
Uric acid (mmol/L)	104.3 ± 28.9	91.3 ± 20.5	**71.34 ± 9.5**	**139.25 ± 64.0^b^**	**104.8 ± 39.6**	**75.6 ± 21.2^a^**
ALT (U/L)	56.8 ± 10.9	80.2 ± 23.4	**66.3 ± 5.1**	**144.0 ± 27.0^b^**	73.5 ± 33.7	83.5 ± 24.9
AST (U/L)	93.8 ± 13.3	91.7 ± 15.3	**156.5 ± 15.0**	**224.2 ± 57.3^a^**	126.7 ± 54.4	122.3 ± 20.4
ALP (U/L)	**330 ± 56.8**	**530 ± 122^b^**	**463.0 ± 64.5**	**1235.2 ± 614^a^**	334.5 ± 89.4	329.5 ± 143
Total protein (g/L)	64.8 ± 3.5	60.8 ± 2.7	65.2 ± 3.2	64.3 ± 7.0	67.7 ± 7.5	68.7 ± 6.4
Albumin (g/L)	**13.7 ± 0.9**	**12.0 ± 1.2^a^**	12.9 ± 0.7	12.8 ± 2.1	13.6 ± 1.3	13.2 ± 1.7
Cholesterol (mmol/L)	1.62 ± 0.1	1.60 ± 0.1	1.72 ± 0.2	1.72 ± 0.5	1.87 ± 0.3	1.69 ± 0.5
Triglyceride (mmol/L)	0.37 ± 0.1	0.36 ± 0.1	**0.97 ± 0.1**	**0.37 ± 0.4^b^**	**1.02 ± 0.4**	**0.42 ± 0.2^b^**
Glucose (mmol/L)	8.32 ± 0.8	7.76 ± 1.0	6.91 ± 0.5	5.37 ± 1.7	8.92 ± 1.3	7.98 ± 0.6

ALT, Alanine aminotransaminase; AST- Aspartate aminotransferase ; ALP- Alkaline phosphatase

Values are expressed as mean ± SD (n= 6 per group).

*P-values were calculated by using the t-test for normally distributed data and the Mann–Whitney test for non-normally distributed data.

^a^ P<0.05; ^b^P<0.01; ^c^P<0.001 as compared to control group.

**Table 4:** Plasma biochemical findings of bacterial cells diet fed and control female rats (7, 14 and 28 days experiments).

Group	7 days		14 days		28 days	
	Control	Test	Control	Test	Control	Test
Calcium (mmol/L)	**2.58 ± 0.1**	**2.30 ± 0.1^c^**	**2.71 ± 0.1**	**2.22 ± 0.1^c^**	2.43 ± 0.10	2.36 ± 0.1
Chloride (mmol/L)	102.2 ± 2.9	105.0 ± 3.2	**103.17 ± 1.5**	**108.00 ± 1.1^c^**	**105.5 ± 1.6**	**98.7 ± 3.7^b^**
Sodium (mmol/L)	135.8 ± 1.7	134.7 ± 2.9	141.83 ± 1.0	140.33 ± 1.8	**135.8 ± 1.5**	**133.8 ± 0.8^a^**
Potassium (mmol/L)	**6.72 ± 0.9**	**5.55 ± 0.3^a^**	4.15 ± 0.4	4.07 ± 0.5	6.27 ± 1.1	6.27 ± 0.4
Phosphate (mmol/L)	2.76 ± 0.3	2.69 ± 0.6	**2.13 ± 0.1**	**1.72 ± 0.3^a^**	**1.89 ± 0.3**	**1.32 ± 0.2^b^**
Creatinine (µmol/L)	**38.6 ± 1.7**	**40.7 ± 1.0^a^**	48.53 ± 2.3	45.32 ± 3.2	43.9 ± 5.5	40.1 ± 1.9
Urea (mmol/L)	**6.98 ± 0.7**	**14.2 ± 2.9^c^**	**6.78 ± 0.5**	**9.38 ± 2.0^a^**	6.98 ± 1.5	7.35 ± 1.8
Uric acid (mmol/L)	103.2 ± 24.3	88.7 ± 20.5	**94.95 ± 17.0**	**157.58 ± 55.0^a^**	74.7 ± 15.8	67.7 ± 12.0
ALT (U/L)	50.8 ± 6.0	52.3 ± 6.3	**47.3 ± 4.8**	**120.5 ± 28.7^b^**	57.3 ± 13.5	66.5 ± 15.6
AST (U/L)	94.5 ± 20.0	85.2 ± 3.8	**159.3 ± 32.8**	**237.8 ± 38.8^b^**	114.2 ± 42.3	121.50 ± 6.7
ALP (U/L)	292 ± 28.8	346 ± 92.6	**298.2 ± 47.3**	**771.3 ± 329.8^b^**	261.2 ± 57.2	224.2 ± 66.6
Total protein (g/L)	66.5 ± 3.6	65.3 ± 2.3	**72.5 ± 2.3**	**65.8 ± 3.9^b^**	76.5 ± 11.4	68.8 ± 4.8
Albumin (g/L)	**14.4 ± 0.9**	**12.7 ± 1.1^a^**	**16.4 ± 0.7**	**13.4 ± 1.7^b^**	15.2 ± 1.5	13.4 ± 1.5
Cholesterol (mmol/L)	1.73 ± 0.4	1.54 ± 0.3	**2.07 ± 0.3**	**1.54 ± 0.3^a^**	1.96 ± 0.3	1.90 ± 0.2
Triglyceride (mmol/L)	**0.65 ± 0.2**	**0.30 ± 0.3^b^**	**0.87 ± 0.2**	**0.43 ± 0.1^b^**	**0.90 ± 0.4**	**0.41 ± 0.1^a^**
Glucose (mmol/L)	9.20 ± 0.5	8.79 ± 0.5	**7.58 ± 0.8**	**5.78 ± 1.6^a^**	**9.03 ± 1.1**	**7.45 ± 0.5^a^**

ALT, Alanine aminotransaminase; AST- Aspartate aminotransferase ; ALP- Alkaline phosphatase

Values are expressed as mean ± SD (n= 6 per group)

*P-values were calculated by using the t-test for normally distributed data and the Mann–Whitney test for non-normally distributed data

^a^ P<0.05; ^b^P<0.01; ^c^P<0.001 as compared to control group.

Interestingly, all the liver function parameters were not statistically significant between control and bacterial cells diet fed animals for 28 days for both sexes. Similar findings were recorded with bacterial cells diet fed animals for 7 days, except only with ALP (*p*=0.004) that showed a significant increase with cells fed male rats. Other than that, serum albumin level was significantly reduced in both males (*p*=0.021) and females (*p*=0.011) given bacterial cells diet. In contrast, significant increases in ALT, AST and ALP levels were detected in both males and females fed bacterial cells diet for 14 days. 

Examination of other biochemistry parameters revealed that the concentration of triglycerides was significantly reduced in all animals given bacterial cells diet except in male rats given this diet for 7 days. Statistically lower serum glucose levels were observed with bacterial cells diet fed females for 14 (*p*=0.032) and 28 (*p*=0.010) days compared to control animals. 

### Organ weights

The relative weights of ileum and kidneys in most of the bacterial cells diet fed animals were found to be statistically higher (data not shown).

### Histopathology

**Figure 3:**
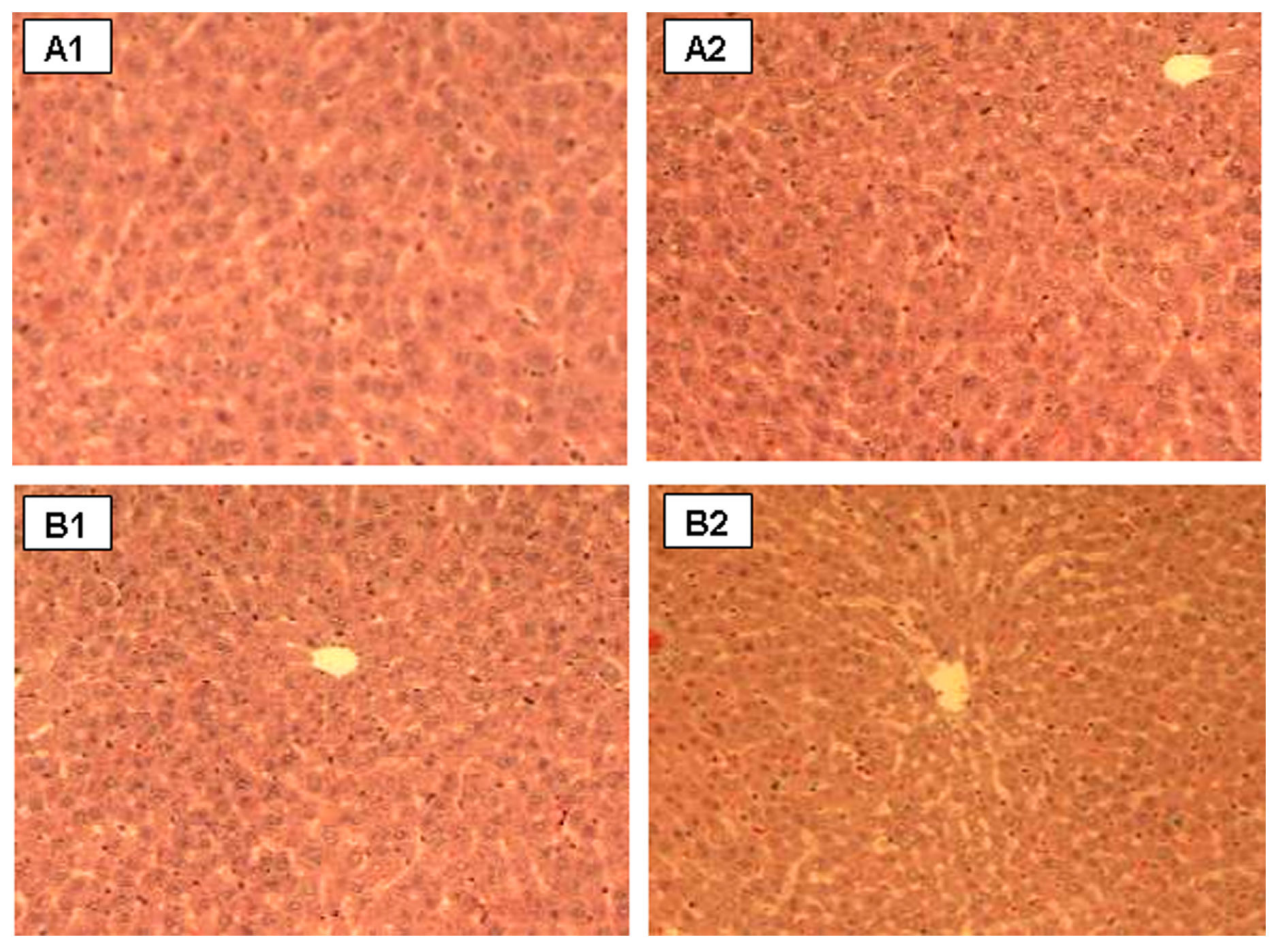
Normal histological appearance of liver sections from control (A1-2) and bacterial cells diet fed (B1-2) animals for 28 days (Magniﬁcation 100×, haematoxylin and eosin stain).

**Figure 4:**
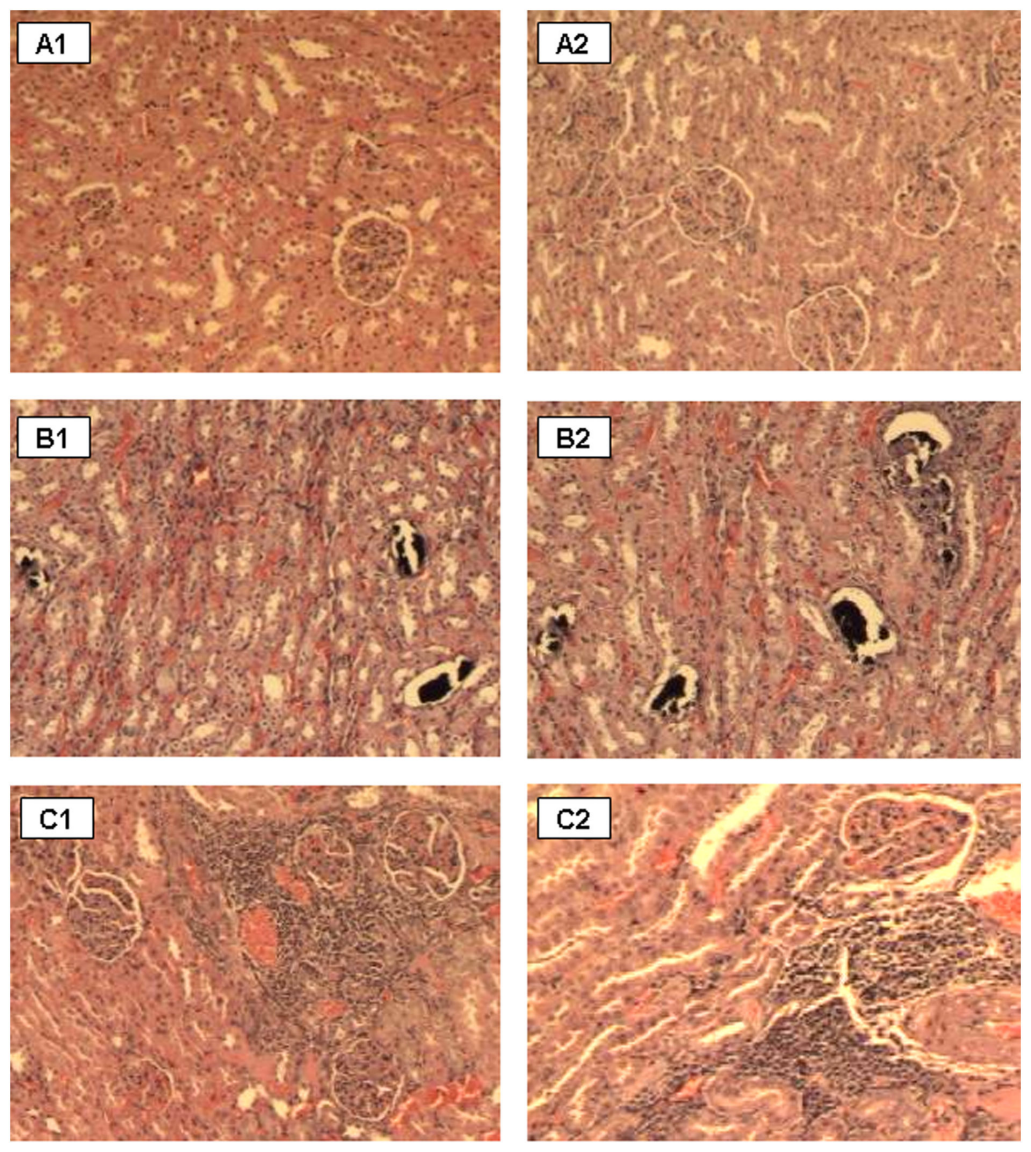
Photomicrographs of kidney sections showing (A) normal histological appearance (B) presence of calcified materials within dilated renal tubules and (C) mononuclear cell infiltrates in the interstitium of Male (A1, B1,C1) and Female (A2, B2,C2) rats given bacterial cells diet for 28 days (Magniﬁcation 100×, haematoxylin and eosin stain).

Normal appearance of liver and kidney were documented in control animal. Histopathology of the liver specimens of all bacterial cells diet fed animals regardless of feeding durations showed no features of cellular damage or inflammation (Figure 3). No pathological alterations of kidney tissues of the experimental animals for 7 and 14 days were detected. Likewise, no diet-related changes were observed in histological sections of the kidney tissues derived from 4 males and 3 female rats receiving bacterial cells diet for 28 days [Figure 4(A1-A2)]. The renal glomeruli and tubular epithelial cells of these animals appeared normal. However renal specimens of the other 2 males and 2 females showed mild to moderate amounts of calcified material within dilated renal tubules [Figure 4(B1-B2)]. A female rat was found to have associated dilated pelvis-calyceal system due to presence of calcified material. Besides, mild to moderate mononuclear cell infiltrates in the interstitium were observed in 3 female rats [Figure 4(C1-C2)]. 

### Viable cell count in excrements

The microbial enumeration determined in the DFP and WFP after being subjected to different pretreatments is presented in Table 5. Higher CFU count (1.4 x 10^7^) was obtained with DFP as compared with WFP (8.4 x 10^3^). However, heating at 60°C significantly reduced both the DFP and WFP viable cell count. Both DFP and WFP that were washed and subsequently heated at 60°C produced fewer than 30 colonies. Morphological analysis through phase contrast light microscopy showed the presence of rod-shaped and motile bacterium which resembled the morphology of *C. necator* H16. Further identification by 16S rRNA is necessary to confirm the identity of this bacterium.

**Table 5:** Effect of temperature and washing pretreatment of fecal pellets on viable cell count.

Type of pretreatments	Raw		Heated at 60°C		Washed and heated at 60°C	
	DFP	WFP	DFP	WFP	DFP	WFP
Viable cell count (cfu/mL)	1.4 x 10^7^	8.4 x 10^3^	6.3 x 10^1^	< 30	< 30	< 30

Readings are means of triplicate.

DFP- dark colored fecal pellets; WFP- white colored fecal pellets; < 30- Less than 30 colonies

### Characterization of the PHB recovered biologically

The WFP was characterized by an outermost layer which was slightly yellowish. Soaking the fecal pellets in distilled water for 24 h removed the yellowish coloration and the protein content of powdered WFP was, at the same time, reduced from 7 ± 1 to 3 ± 1 wt%. This showed that the washing procedure was effective in removing some of the fecal proteins, including, presumably, those present in the outer yellowish layer. GC analysis confirmed the presence of PHB in WFP (Table 6). PHB content of the outermost yellowish layer of the WFP was 82 ± 3 wt%, while the layer beneath this yellowish coating had an increased PHB content of 94 ± 1 wt%. The highest PHB content (97 ± 3 wt%) was found towards the center of the fecal pellet. Slightly higher PHB content was obtained from the surface (95 ± 1 wt%) and in the center (98 ± 2 wt%) of the washed WFP samples.

**Table 6:** Thermal characterization of the PHB granules obtained using biological and solvent extraction method.

Sample	PHA content (wt%)	*T*_m_^a^ (°C)	*T*_g_^b^ (°C)	Δ*H*_m_^c^ (J/g)	X_c_^d^ (%)
Untreated Pellet (Yellow layer)	82 ±3	176	1.0	67	46
Untreated Pellet (surface)	94 ±1	175	1.5	78	53
Untreated Pellet (centre)	97 ±3	174	6.8	78	53
Treated Pellet (surface)	95 ± 1	175	3.0	85	58
Treated Pellet (centre)	98 ± 2	176	4.5	89	61
Freeze-dried cell	39 ± 1	167	-8.0	89	61
PHB (solvent extraction)	99 ± 1	173	2.0	79	54

For PHA content, data shown are the means of triplicate. Treated Pellet-Washed using water and heated at 60ºC. ^a^Melting temperature; ^b^Glass transition temperature; ^c^Heat of fusion; ^d^Degree of crystallinity

### Molecular mass of PHB

PHB synthesized by *C. necator* H16 was extracted using chloroform. SEC analysis showed the weight average molecular weight (*M*_w_) to be 950 kg/mol based on polystyrene standards indicating that PHB synthesized by *C. necator* H16 using CPKO as the sole carbon source was of a relatively high molecular mass. Biologically extracted PHB granules were determined to have a *M*_w_ of 930 kg/mol. Further purification of the granules with water did not cause significant reduction in their *M*_w_ (770 kg/mol).

### Differential scanning calorimetry (DSC)

The thermal properties (Table 6) of the PHB samples recovered biologically and samples extracted using solvent were determined using DSC. The melting temperature (*T*_m_) of both untreated and treated samples appeared to be in a narrow range of 174-176°C, which was within the range of 173-180°C reported in the literature [9,33]. *T*_m_ of 173 °C was obtained with the PHB recovered by solvent extraction. The degree of crystallinity, which is a critical factor in regulating mechanical properties, was calculated by assuming the heat of fusion (Δ*H*_m_) for 100% crystalline PHB was 146 J/g [34]. The percentage of crystallinity (X_c_) for the PHB granules recovered biologically (46-61%) did not show much deviation from the value obtained with PHB isolated using solvent extraction (54%) when taking into account the mass of non-polymeric constitution. 

Comparison of the recovery efficiency of the newly developed biological recovery process with different PHA extraction methods 

Acids, alkaline, surfactants and strong oxidizing agents are commonly employed in the chemical digestion methods for PHA recovery [8]. Therefore, HCl, NaOH, SDS and NaOCl were selected to represent each group respectively and their concentrations were varied in order determine the effects on the polymer purity and yield (Table 7). A method that takes advantage of both chloroform and sodium hypochlorite was also investigated [35]. As expected, polymers recovered by solvent extraction exhibited a higher level of purity (97 wt%) with slightly lower recovery yield. A comparison made between solvent and biological extraction revealed that both methods resulted in relatively similar purities and yields. Polymers of higher purities (91-96 wt%) were also recovered with dispersion of chloroform and sodium hypochlorite solutions of different concentrations. However, the polymer yields were determined to be significantly affected by this technique (34-50 wt%). HCl (53-58 wt%) was found to be inefficient for the recovery of polymer with high purity. Increasing the HCl concentrations did not improve the P(3HB) purity. Slightly better polymer purities were noted with higher concentrations of NaOCl (66-70 wt%) though the values remained relatively constant with increasing solution strengths. Digestions with higher concentrations of NaOH (83-87 wt%) and SDS (77-88 wt%) contributed to better polymer purities. The recovery yield of P(3HB) from cells digested with HCl (90-93 wt%) and NaOH (84-92 wt%) of different concentrations were comparatively higher than the solvent extraction. On the other hand, SDS (62-76 wt%) and NaOCl (71-86 wt%) gave recovery yields almost in the range as solvent extraction. From these findings, it can be concluded that recovery efficiency of biological extraction is comparable with the solvent extraction method. In general, the purity of P(3HB) recovered biologically was more superior than the chemically digested polymer. The polymer yields were still in the acceptable range when compared with chemical digestion methods. 

**Table 7:** Comparison of the PHA recovery efficiency from *C. necator* H16 using solvent extraction, biological extraction and digestion with various chemicals.

Treatment	Purity (wt%)	Yield (wt%)
Solvent extraction^a^	97 ± 3^p^	72 ± 4^p^
Biological extraction^b^ (Treated Pellet at surface using water)	96 ± 2^p^	74 ± 3^p^
Digestion with HCl^c^ (M)		
0.05	53 ± 9^r^	91 ± 2^r^
0.1	57 ± 5^r^	91 ± 2^r^
0.2	58 ± 7^r^	90 ± 3^r^
0.5	57 ± 1^r^	93 ± 1^r^
Digestion with NaOH^c^ (M)		
0.05	74 ± 4^s^	84 ± 4^ps^
0.1	83 ± 5^st^	90 ± 6^s^
0.2	87 ± 3^st^	92 ± 1^s^
0.5	84 ± 3^t^	85 ± 5^ps^
Digestion with SDS^c^ (g/L)		
0.5	54 ± 5^u^	62 ± 4^t^
2.5	77 ± 6^v^	64 ± 5^pt^
5.0	86 ± 4^pv^	76 ± 7^pt^
10.0	88 ± 5^pv^	73 ± 5^pt^
Digestion with NaOCl^c^ (% v/v)		
5	53 ± 6^w^	86 ± 2^p^
10	66 ± 2^x^	71 ± 9^p^
15	70 ± 9^x^	78 ± 8^p^
20	69 ± 1^x^	84 ± 3^p^
Digestion with NaOCl (% v/v) and Chloroform^c^ (ratio of 50:50)		
5	93 ± 2^p^	37 ± 2^xy^
10	91 ± 7^p^	34 ± 6^x^
15	96 ± 2^p^	48 ± 7^yz^
20	95 ± 4^p^	50 ± 3^z^

HCl, hydrochloric acid; NaOH, sodium hydroxide; SDS, sodium dodecyl sulphate; NaOCl, sodium hypochlorite; CHCl_3,_ chloroform

a Extraction using reflux method by mixing 1 g of lyophilized cells with 100 mL of chloroform followed by precipitation with methanol.

b Purification of biologically extracted P(3HB) granules by mixing 1 g of the fecal pellets in 5 mL of distilled water.

c Digestion of 1 g of lyophilized cells in 100 mL of chemical solution at 30°C and 250 rpm for 1 h

Data shown are means of triplicate experiments. Mean values indicated by different superscript alphabets are significantly (Tukey’s HSD test, p<0.05).

## Discussion

Bacterial cells are a well known source of protein [18]. Bacterial proteins featured prominently in the diet of *C. necator* H16 cells in the present study. These bacterial cells are also identified as a source of trace elements which are sometimes added to the culture to promote PHA biosynthesis [24]. In general, the present study revealed that these animals were able to survive solely on the bacterial cells diet containing moderate amount of PHA up to one month, though they grew at a much slower rate than their respective controls. It has been reported that feeding rats a moderately high protein diet results in lean body mass as well as reduction in the fat mass [36]. Although the average daily food intake of animals given bacterial cells diet was similar to controls receiving commercial diet, the bacterial cells diet fed animals exhibited poor weight gain due to the fact that the actual nutritious portion of the feed received by these animals was less than half by weight as compared with the commercial feed consumed by control animals. Further studies are required to address a diet formulation that balances both animal’s growth and PHA excretion in their fecal pellets. The animals fed with bacterial cells diet for longer durations did not exhibit any signs of diarrhoea. The breakdown of PHB into absorbable monomers such as 3-hydroxybutyric acid is claimed to be useful for treating or preventing diarrhoea [24]. 

 The water intake of bacterial cells diet fed rats was approximately two-fold higher than the controls. It is known that water requirement in animals fed with high protein diet is elevated, since large amounts of urea must be excreted via the urine [37].

 The concentrations of red blood cells, haemoglobin and haematocrit are interrelated and typically affected by protein intake. Diet high in protein improves the synthesis of red blood cells and haemoglobin due to better bioavailability of amino acids that make up this cellular type [38]. The increase in neutrophil concentrations of bacterial cells diet fed rats is commonly associated with a response to stress [39]. According to Selye [40], many types of dietary deficiencies can also act as stressor agents in exerting a pronounced effect on the blood counts.

 In contrast to the fact that food high in protein elevates the concentration of serum phosphorus [41], lower amount of serum phosphorus was detected with all bacterial cells diet fed animals. Protein sources contain varying amounts of phosphorus, and the bioavailability of this phosphorus may not be the same for all sources [29]. The unbalanced Ca/P ratios have been identified to inhibit calcium absorption [42]. A decrease in albumin level was noted in bacterial cells diet fed animals implying a decrease in protein bound calcium level [43]. Normally pronounced absorption of sodium, potassium and chloride would be expected with increased water intake by animals given high protein diet [37]. However, in the present study, the differences between serum sodium, potassium and chloride concentrations of animals given bacterial cells diet with their respective controls were random regardless of feeding durations though water consumption of experimental rats was higher. The observed effects could be attributed to some inherent imbalances in the mineral composition of SCP proteins. Typically, the functions of minerals are interrelated. When a mineral is taken in excess or insufficient quantity, it can influence the intestinal absorption of other minerals [43].

The elevation in serum urea nitrogen was documented with animals receiving bacterial cells diet compared to controls. Degradation of nitrogenous substances in the bacterial cells diet such as pyrimidines from the nucleic acids and increased excretion of protein waste are known to increase serum urea nitrogen [39]. The main factor that hinders the application of microbial protein products is the presence of high nucleic acid content. However, nucleic acid is not a toxic component and it causes only physiological effects when taken excessively like any other essential dietary ingredients. Dietary nucleic acids have great influence on the blood uric acid concentration. High levels of uric acid in the body can result in the development of kidney stones or gout [44-46]. Uric acid is the end product of purine metabolism in mammals that lack of uricase enzyme. It has been highlighted previously that lower forms of animals such as rats possess a full complement of enzymes necessary for degrading uric acid [47]. No kidney stones were observed with histological sections of the kidney tissues of rats treated for 14 days despite high uric acid concentrations. In contrast, test animals given bacterial cells diet for 7 and 28 days demonstrated normal uric acid levels when compared to controls. Hence, it could be inferred that increase in uric acid level is merely a response to metabolic requirements while the rats developed adaptive mechanism when fed with bacterial cells diet for longer duration. 

Normally, the level of aminotransaminases in blood serum will increase when body tissue or an organ such as the liver or heart is diseased. Liver histopathology of animals given bacterial cells diet for 14 days did not show any features of cellular damage or inflammation that could be associated with increases in ALT and AST activities. Besides, the association between high levels of serum ALP and liver disease also has been widely known [48,49]. Elevation in the level of ALP was detected in treatment animals given bacterial cells diet for 7 and 14 days in the absence of any histopathological lesions in the liver tissues. Since, the enzyme is responsible for removing phosphate groups from many types of molecules such as DNA, RNA, nucleotides and proteins [48]; hence the presence of high nucleic acids in the bacterial cells diet in addition to the high protein content could be linked to the increase in ALP activity. Metabolic adjustment to a high protein diet has been correlated with increase in the activity of enzymes involved in protein digestion and in splanchnic nitrogen metabolism [36,50].

 The bacterial cells diet is very low in carbohydrates correlating with the alteration in glucose level of treatment animals. Most of the hydrogen bacteria including *C. necator* H16 are known to store lipid inclusion in the form of PHA while only a few strains are found to produce triglycerides, and others polysaccharides [18]. The possible use of PHB by animals for nutritional purposes has not been established [17,20,21,23,24]. 

 Comparing the organ weights of the bacterial cells diet fed animals with controls may reveal specific organ changes related to the diet [51]. As the body weights of bacterial cells diet fed animals was observed to be lower than controls, hence relative organ weight was determined by taking the ratio of the organ weight to body weight. Relative kidney, ileum and stomach weights of bacterial cells diet fed animals were higher than controls. As the monomer 3-hydroxybutyric acid is known to be well absorbed, the PHB biopolymer has been highlighted to be poorly digested in the gastrointestinal tract of animals [17,20,21,23,24]. The retention of the ingested PHB in the ileum and stomach of treated animals can be correlated with the differences in relative organ weight between bacterial cells diet fed and control animals. The mineral deposition could partly cause the increase in relative kidney weight. 

Liver and kidney are important organs in toxicity studies and are especially vulnerable to damage. No positive relationships between biochemistry and histopathological of liver could be derived from all the tolerability and safety evaluation experiments. This could be due to the nutritional status of the bacterial cells diet. The discrepancies in biochemical parameters of renal functions of animals given bacterial cells diet for 7 and 14 days could not be associated with kidney damage but merely as response to metabolic requirements. However, mild to moderate diet-related changes in the kidney histopathology were found with four rats receiving bacterial cells diet for 28 days that could be linked with serum markers of renal injury. The presence of mild to moderate amounts of calcified material within dilated renal tubules of two males and two females could be related to the unbalanced Ca/P ratio in the bacterial cells diet [43]. It could be inferred that the detection of potentially diet-related renal effects and morphological changes in some of these animals may be an early indicator of the nutritional status of the high protein bacterial cells diet. It is also possible that feeding for 28 days may not be long enough to cause sufficient treatment-related histopathological changes of both liver and kidney to accompany alterations in clinical biochemistry associated with liver and renal functions.

The detection of high PHA content in the WFP can be explained by the excretion of PHB originally derived from the bacterial cells diet. To date, there is no report of PHB being metabolized by animals into an energy source [17,20,21,23,24]. The bacterial cell wall is apparently not resistant to the digestive enzymes of the intestinal tract of rats [17]. The poor digestibility of PHB could be associated with the absence of depolymerase producing microorganisms in the animal’s gastrointestinal tract [20,21,23,24]. Furthermore, the rapid passage of PHB granules in animals’ digestive system and relatively high crystallinity of PHB could limit its degradation [20,21,23,24,52]. Though higher molecular weight PHB granules are associated with poor digestibility, there is a possibility of the biopolymer breaking down into shorter chain lengths or into absorbable monomers such as 3-hydroxybutyric acid [20,21]. It was claimed that less than 10% of bacterial PHB could be digested in rats [17]. The possible presence of PHB in the amorphous state may also be attributed to higher degradation rates [23]. In fact, the breakdown of PHB into 3-hydroxybutyric acid, which is an important intermediate in cellular metabolism offers some advantages [18,53]. 

It can be seen that microbial lower viable cell count was obtained with fecal pellets of test group regardless of the pretreatment. The washing steps employed, including the heat treatment at 60°C, were aimed at eliminating almost all living microorganisms in the fecal pellets. However, safety assessment of biologically recovered PHB needs to be established. The low levels of protein found in powdered WFP indicated that the protein component of the bacterial cells diet was well digested and absorbed by the test animals. This is a desirable sign, affirming the nutritive value of the bacterial cells diet as an animal feedstock while the PHB biological recovery process is in operation. 

Since it has been acknowledged that solvent extraction causes negligible degradation of PHB, values obtained with SEC were assumed to be the intact molecular weight. It is important to emphasize that PHB granules recovered biologically possessed similar *M*_w_ compared to chloroform extracted PHB. This finding revealed that PHB was not degraded in the animal model digestive system [20,21,23,24]. Besides, SEC measurements revealed that purification procedure of biologically extracted PHB did not induce any big differences in the molecular mass and their distribution. DSC analysis showed that thermal properties of biologically recovered PHB are comparable with PHB extracted using chloroform. 

 The fecal pellets containing PHB can be obtained from rats as early as the following day after feeding the rats. About 350 g of WFP were collected with just 12 animals in a week, indicating that large amounts of biopolymers could be recovered efficiently within a short period. The quantities of WFP obtained during 2 and 4 weeks treatments were found to be approximately 3 to 5 fold higher with respect to one week collection. The amount of fecal pellets collected can be varied depending on the feed consumption rate and number of the rats. The entire recovery process of PHB described in this paper did not involve the use of any solvents.

 Chemical digestion methods are well established approaches developed as an alternative to solvent extraction. These chemicals facilitate PHA recovery by hydrolyzing the non-PHA cell mass (NPCM), including the peptidoglycan of cell walls, thus releasing proteins and other biological macromolecules into the aqueous solution [7]. In the present study, relatively similar or higher PHA yield was obtained with almost all the digestion methods except using dispersion of chloroform and sodium hypochlorite when compared to biological extraction. Polymers with reasonable or lower purities were isolated with most of the chemicals except dispersion of chloroform and sodium hypochlorite that yielded P(3HB) of high purity as biological extraction.

 In the case of HCl digestion, acid strength and temperature are important factors for high recovery efficiency; however process parameters have to be controlled stringently if the molecular weight is to be maintained at a minimum of 50% of the original molecular weight. Besides, the P(3HB) granules recovered by this method were reported to be highly crystalline [7]. The important features of sodium hypochlorite such as strong oxidizing properties and non-selectivity can be manipulated to digest NPCM and facilitate PHA recovery but it also known to promote severe degradation of molecular weight at higher concentrations [8]. Although polymer of high purity can be obtained using dispersion of chloroform and sodium hypochlorite, the main limitation is that it still involves the use of large quantities of toxic and volatile solvents [14].

 The P(3HB) purities can be improved by increasing NaOH and SDS concentrations. However, it is crucial to highlight that alkaline conditions also cause hydrolysis of (P3HB) [7]. Since the use of surfactant alone cannot isolate PHA of high purity, other agents such as hypochlorite and NaOH are needed [14]. In addition, though recovery of PHA using surfactant is technically simple, however, concentration above 5 wt% may cause problems in wastewater treatment and increase the recovery cost. Better PHA recovery yield and purity from *C. necator* cells have been documented when using aforementioned chemicals in previous studies [7,54,55]. The recovery yield and purity of PHA of an extraction method is strongly dependent on PHA content of the cells. Generally, cells with lower PHA content often result in a higher recovery cost as larger amount or higher strength of digesting agents are needed for extraction of the polymer simultaneously increasing the cost of waste treatment [56]. It is important to highlight that regardless of the low PHA content of the cells; PHA of high purity can still be isolated using the biological extraction method. 

 Although the newly developed biological recovery method offers many advantages, the applicability to large scale usage is still questionable. Cultivation of large number of laboratory rats primarily for this purpose needs to be viewed carefully as rats are effective agents of disease transmission. Besides, the possibilities of using other animals such as ruminants for recovery of PHA need to be investigated. Social acceptance is another underlying problem with this newly developed method. The use of biologically extracted polymers to replace conventional plastics in certain applications is dependent on the consumer’s acceptance. It is important to emphasize that the biologically recovered polymers are targeted for agricultural applications. 

## Conclusions

The findings of the present research demonstrate that *C. necator* H16 cells can be considered as a protein source for rats. However, further studies are required concerning formulation of diet with protein source from the bacterial cell that meets nutritional requirements of rats in order to overcome the issues such as poor growth and potential development of kidney stones. The present study has also contributed positively towards the development of a novel biological recovery method of PHA. Biologically recovered PHA may be applicable in products where high level of purity is not necessary such as for the making of biodegradable mulching films and slow release fertilizers.
